# Predicting the Quality of Meat: Myth or Reality?

**DOI:** 10.3390/foods8100436

**Published:** 2019-09-24

**Authors:** Cécile Berri, Brigitte Picard, Bénédicte Lebret, Donato Andueza, Florence Lefèvre, Elisabeth Le Bihan-Duval, Stéphane Beauclercq, Pascal Chartrin, Antoine Vautier, Isabelle Legrand, Jean-François Hocquette

**Affiliations:** 1 UMR Biologie des Oiseaux et Aviculture, INRA, Université de Tours, 37380 Nouzilly, France; elisabeth.duval@inra.fr (E.L.B.-D.); s.beauclercq@gmail.com (S.B.); pascal.chartrin@inra.fr (P.C.); 2 UMR Herbivores, INRA, VetAgro Sup, Theix, 63122 Saint-Genès Champanelle, France; brigitte.picard@inra.fr (B.P.); donato.andueza@inra.fr (D.A.); jean-francois.hocquette@inra.fr (J.-F.H.); 3 UMR Physiologie, Environnement et Génétique pour l’Animal et les Systèmes d’Élevage, INRA, AgroCampus Ouest, 35590 Saint-Gilles, France; benedicte.lebret@inra.fr; 4 Laboratoire de Physiologie et Génomique des poissons, INRA, 35000 Rennes, France; florence.lefevre@inra.fr; 5 Institut du porc, La motte au Vicomte, 35651 Le Rheu, CEDEX, France; antoine.vautier@ifip.asso.fr; 6 Institut de l’Elevage, Maison Régionale de l’Agriculture—Nouvelle Aquitaine, 87000 Limoges, France; isabelle.legrand@idele.fr

**Keywords:** meat, quality, prediction, biological marker, spectroscopy, phenotypic model

## Abstract

This review is aimed at providing an overview of recent advances made in the field of meat quality prediction, particularly in Europe. The different methods used in research labs or by the production sectors for the development of equations and tools based on different types of biological (genomic or phenotypic) or physical (spectroscopy) markers are discussed. Through the various examples, it appears that although biological markers have been identified, quality parameters go through a complex determinism process. This makes the development of generic molecular tests even more difficult. However, in recent years, progress in the development of predictive tools has benefited from technological breakthroughs in genomics, proteomics, and metabolomics. Concerning spectroscopy, the most significant progress was achieved using near-infrared spectroscopy (NIRS) to predict the composition and nutritional value of meats. However, predicting the functional properties of meats using this method—mainly, the sensorial quality—is more difficult. Finally, the example of the MSA (Meat Standards Australia) phenotypic model, which predicts the eating quality of beef based on a combination of upstream and downstream data, is described. Its benefit for the beef industry has been extensively demonstrated in Australia, and its generic performance has already been proven in several countries.

## 1. Introduction

The control of meat quality, especially sensory traits, remains an important issue for any farm animal production. This is the case for ruminant meat, in particular beef, but also for poultry and pork, although, for them, controlling the technological quality (processability) is at least as important [1]. Research efforts over many years, particularly in Europe, have led to a better understanding of the impact of the various production factors on the muscle characteristics and the quality of the meat obtained. At the same time, it highlights the complex determinism of the biological characteristics of muscles and meat, which is most often driven by many factors related to genetics as well as animal husbandry and slaughter systems [2]. Under these conditions, having reliable indicators to predict meat quality is a major challenge for the meat industry. These indicators would facilitate the selection of animals that are capable of producing good quality meat. In addition, they would improve breeding and slaughtering practices with the aim of optimizing the intrinsic qualities of muscles. With this in mind, many studies have explored the possibilities offered by different methodologies in the fields of biology, physical chemistry, and modeling to predict meat quality. This review aims to present, through examples, the different approaches developed in Europe (with the exception of genetic tests) to better predict the sensory and technological quality of meat and meat products. It also provides a critical analysis, in the light of the results obtained or the obstacles identified, to the deployment of these new tools at the industrial level.

## 2. Seeking the Genes that Control the Quality of Pork and Chicken Meat

Pork and poultry are the two most widely consumed meats in the world, in the form of a wide variety of products, both fresh and processed. Therefore, the notion of meat quality in these species is complex, and the technological and sensory traits, which have often common determinants, are generally considered simultaneously to evaluate product quality [3,4]. As with all species, the quality of poultry and pork is the result of interactions between genetic, breeding, slaughtering, and processing factors. Although many genetic or breeding factors influencing meat quality have been identified, this complex phenotype remains variable and difficult to predict. Therefore, the search for quality ante- or post-mortem biomarkers has started in these species in order to predict—in vivo or quickly after slaughter—the technological or sensory quality of meat or carcasses to optimize their use in different processing sectors. There are many potential applications: the selection of breeding animals but also, in the case of pork, the sorting of carcasses or parts of carcasses at the slaughterhouse (reviewed in [5]). In these two species, several studies have sought to find muscle transcripts for meat quality prediction.

### 2.1. Biomarkers of Meat Quality in Pork

In pork, the identification of meat quality biomarkers through transcriptomic approaches was performed on the loin and ham muscles. Studies focusing on the main sensory or technological traits of pork were carried out on different animal models, including experimental groups with extreme intramuscular fat (IMF) content, shear force value of cooked loin, pale, soft, exudative (PSE) defect, or affected or not by the presence of ‘destructured’ (also called “PSE-like”) zones found especially in the *Semimembranosus* muscle. This quality defect is a major issue that seriously impairs the processing yield and the sensory quality of cooked ham.

In 2006, Damon et al. [6] identified the first molecular markers of ‘destructured’ ham muscle. They included several genes encoding the myofibrillar proteins involved in actin–myosin interactions and sarcomere integrity (tropomodulin, ankyrin, myomesin) and the enzymes involved in the glycolytic pathway. In pork, IMF is an important phenotypic trait that contributes to the tenderness and juiciness of meat. Liu et al. and Hamill et al. [7,8] showed that the IMF content of the *Longissimus* muscle (loin) at the slaughter stage is associated with the expression of genes involved in different functions: carbohydrate and lipid metabolism, cell communication, binding, response to stimulus, cell assembly, and organization. These results strengthen the fact that IMF content depends on the regulation of various metabolic and cellular pathways. These studies also highlighted the significant role of genes involved in adipogenesis regulation during animal growth (70 kg live weight) determining the IMF content at slaughter (110 kg). This reinforces the hypothesis that the inter-individual variability in IMF content depends on the early expression of genes regulating the development of intramuscular adipocytes [7]. Overall, this explains the difficulty in finding robust biomarkers of IMF content that can be measured from muscle samples taken at slaughter. 

Several studies aimed to find predictive biomarkers of loin tenderness, assessed either by a trained sensory panel or by measuring the Warner–Bratzler shear force (WBSF) of cooked samples. The microarray transcriptomic profiles of loin muscle varying in WBSF were compared, and classification or regression analyses were used to identify 63 genes strongly associated with WBSF [9,10]. Integration of these transcriptomic results with proteomic data obtained from the same samples [11] showed that low WBSF values (i.e., tender meat) are associated with genes involved in the regulation of lipid metabolism, while high WBSF (i.e., tough meat) is related to genes controlling the morphology of muscle fibers (number, size, sarcomere, etc.). Furthermore, analysis of the transcriptomic data using random forest methodology identified 12 genes that are most important in determining the tenderness of cooked loin [9,10].

Since the sensory and technological qualities of pork are complex phenotypes that are determined by different traits (color, pH, drip loss, marbling or IMF, tenderness, juiciness, etc.), further studies aimed at identifying predictive biomarkers of pork quality simultaneously considered the variability of several meat quality traits within or between breeds or between production systems. Furthermore, and contrary to what has been done before, these studies included further validation steps to test the generic biomarker prediction on additional meat samples taken from the same population used for their identification as well as others. For instance, using an experimental design involving two breeds produced in different farming systems, it was possible to generate gradual and high variability in the technological and sensory qualities of pork [12]. This set of animal samples was used to identify and further validate biomarkers of pork quality. The muscle transcriptome profiles of 50 loins sampled 30 minutes after slaughter were associated with several meat quality traits: ultimate pH, drip loss, lightness (L*), redness (a*), hue angle (h°), IMF, as well as the WBSF, tenderness, and juiciness of the cooked meat. The wide range of meat traits and gene expression patterns made it possible to establish thousands of correlations between gene expression and meat quality traits (140 for a* up to 2892 for tenderness). Then, 40 genes selected for their high correlation coefficient value or relevant biological process terms regarding muscle development and meat quality were considered for a first “technical” validation step from the same 50 loin samples, assessing gene expression by real-time PCR, which is a sensitive and fast method that can be used when developing molecular diagnosis tools. At this step, 113 transcript-trait associations were confirmed, of which 60 were further validated (*R²* ≤ 0.46, *p* < 0.05) for eight meat quality traits using 50 additional animals from the same experimental design. This means that among the genes identified, the level of expression of one gene could explain up to 46% of the variability of one meat quality trait [13]. Finally, an external validation was carried out on 100 commercial pigs (Duroc × Landrace × Yorkshire), validating 19 of these biomarkers (*R²* ≤0.24, *p* < 0.05) that were correlated to the ultimate pH of meat (6), drip loss (4), L * (5), h° (2), IMF content (1), and tenderness (1) [14]. In addition to single correlations, multiple regression models were calculated from the quantification of gene expression by real-time PCR. Models with three to five genes explained up to 59% of the phenotypic variability of meat quality traits. The best accuracy (highest *R²*) was found for meat color (h°), ultimate pH, drip loss, and IMF [13], but their predictive value when tested on commercial pigs (Duroc × Landrace × Yorkshire) was quite moderate (*R²* ≤ 0.23, *p* ≤ 0.01 between the predicted and measured value).

Until now, several biomarkers of individual pork quality traits have been identified, and some have been validated in different pig populations. However, their predictive capacity still needs to be improved before considering using for diagnosis purposes in the pork industry. Therefore, another strategy was considered. It aims to identify and validate biomarkers of a meat quality level combining both sensory and technological dimensions (instead of predictors of single meat quality traits), with the ultimate objective of proposing molecular tools to classify carcasses or primary cuts soon after slaughter in the meat industries, according to their predicted quality level. Using the meat quality data for pork loin samples from the experimental design presented above (two breeds and production systems, *n* = 100) and combining scientific expertise and statistical approaches, three classes differing in both sensory and technological qualities were specified: low (defective), acceptable, and extra. The expression levels (qRT-PCR) of 40 genes previously obtained on these 100 loin samples were used as predictive variables in a generalized linear model (stepwise selection) to discriminate quality classes. The best predictive model included 12 genes corresponding to different biological functions associated with meat quality development: mitochondrial energy metabolism, lipid and carbohydrate metabolism, gene expression control, cell regulation and apoptotic processes, calcium transport, protein transport, muscle structure and contraction, and muscle hypertrophy. After cross-validation using the leave-one-out method, this model exhibits an overall correct classification rate of 76%, with 88% for defective samples and 82% for extra samples [15]. 

### 2.2. Biomarkers of Meat Quality in Chicken

In chicken, several studies have sought to find muscle transcripts that predict the quality of the breast meat. They took advantages of several experimental animal designs. These are groups of individuals with extreme muscle characteristics (acid meats or DFD for “dark, firm, dry”) from a single population of divergent lines selected for body composition or meat quality, or cross-breeds between lines to access individuals who are extreme in term of meat quality, but have a homogeneous genetic background for other traits such as production. The main traits studied were related to the post-mortem (p.m.) pH drop, in particular the pH measured at 15 min p.m. (pH15) and the breast meat pH measured at 24 h p.m. (ultimate pH, or pHu). Both influence the physicochemical and functional properties of proteins and affect a large number of quality parameters: color, water-retention capacity, hardness after cooking, technological yield, and susceptibility to oxidation [16,17,18].

The first network of genes identified as being related to the breast meat pHu was obtained by studying lean or fat chicken lines. In chicken, selection for low carcass fattening has led to changes in muscle properties: lean animals have lower glycogen reserves than fat animals, resulting in a higher meat pHu and better technological yield [19]. The results highlighted the involvement of several important pathways for glycogen control in muscle, such as the AMP dependent pathway involving the AMP-activated protein kinase (AMPK) complex as well as the cyclic AMP-dependent signaling pathways, and pathways involved in the control of carbohydrate availability in muscle [20]. To overcome the demonstrated links between peripheral fattening and glycogen storage ability in chicken muscle [19], a divergent selection based on a modern commercial broiler line was made, allowing the creation of two divergent lines specifically for breast pHu [17]. Transcriptome analysis of the breast muscle revealed very different metabolic statuses and energy production modes between these two lines (pHu+ and pHu−). The pHu of the breast muscles mainly use their high reserve of carbohydrate, while those of the pHu+ line use alternative catabolic pathways leading to significant remodeling of the muscle tissue [21,22]. From the transcriptome, including 1436 genes identified as differential between pHu+ and pHu− individuals, sPLS (sparse partial least squares) models were adjusted to predict pHu. The fitted models have good explanatory and predictive ability of the pHu (R²Y = 0.77–0.87, Q² = 0.68–0.79). Twenty-one genes from this model supplemented by 27 other biomarker candidate genes were selected for high-throughput qRT-PCR validation (Fluidigm technology) in a population of 280 animals from both lines (pHu range 5.41–6.50). After a step of elimination of the genes with low explanatory abilities, a final PLS model including 20 confident genes was adjusted, which could be used to predict the pHu of the breast meat with an explanatory power (R^2^) of 0.65, a predictive power (Q^2^) of 0.62, and an error rate of 16% [22].

Recently, studies carried out on animals with pedigree information have made it possible to combine positional (quantitative trait loci, QTL) and expressional (transcribed) data with two objectives: identifying the genetic markers or mutations responsible for the variation of meat quality traits and facilitating the identification of fine molecular phenotypes for diagnosis and selection purposes. This strategy has already demonstrated its effectiveness in a study on the color of chicken meat. The detection of an expression QTL (or eQTL) confirmed that the gene BCMO1 (which encodes β-carotene 15,15′-monooxygenase 1) was responsible for variations in the yellow color of chicken meat and accelerated the identification of causal mutations within its promoter region [23]. These results led to the development of a patented genetic test [24] currently available to breeders who wish to control the yellow color of chicken breast meat in response to variations in the composition of feedstuffs. Studies on the interactions with feed have demonstrated the possibility of modulating the deposition of xanthophyll pigments and therefore the coloration of meat through this test [25,26]. Recently, a similar approach took advantage of two broiler lines divergently selected for the ultimate pH of the pectoralis major muscle to decipher the genetic control of this trait. By combining the detection of selection signatures and QTL with whole transcriptome analysis, it has identified genomic regions and a major candidate gene for chicken breast meat ultimate pH: PPP1R3A. It codes for a muscle-specific regulatory subunit of protein phosphatase 1 (PP1) that promotes the dephosphorylation of glycogen synthase (GS) and glycogen phosphorylase (GP), and thus glycogen synthesis. It was differentially expressed between the pHu+ and pHu– lines [22] and was located close to the most significant single-nucleotide polymorphism (SNP) for pHu [27].

These promising results (listed in Table 1) make it possible to foresee the development of tools to sort animals or carcasses based on their quality level. Indeed, while it is unlikely that diagnostic tests based on gene expression measurement will be used commercially, the identification of these intermediate molecular phenotypes will facilitate the future development of more accessible and/or less invasive techniques that are useful for breeding or industrial purposes. 

## 3. Quantification of Proteins to Predict the Tenderness of Beef

One of the main objectives of beef research has long been to control and predict meat tenderness. Although many studies have clarified the role of muscle components such as muscle fibers, connective tissue, lipids, proteases [28], and the well-documented effects of muscle type, animal type or breed, and meat aging [2], meat tenderness remains poorly controlled by the beef industry. Currently, the techniques available to evaluate this trait are mechanical measurements, sensory analysis by trained panels, or consumer tests that are generally performed after meat aging and on cooked meat. These methods are quite complex, time-consuming, and expensive to apply, and the meat industry is still waiting for objective criteria and tools to evaluate and predict meat tenderness in live animals or quickly after slaughter to improve carcass valuation and limit consumer dissatisfaction.

One strategy implemented over the past 10 years has consisted of identifying protein biomarkers of tenderness with the objective of proposing a molecular test to evaluate beef tenderness [5,29,30]. Protein analysis provides additional information that gene or transcript analysis cannot give, because the expression of a gene does not always mean that the corresponding protein is proportionally expressed in the tissue of interest. Moreover, a protein can exist in several forms (isoforms) and can undergo post-translational modifications. This can only be observed at the protein level after separation by two-dimensional electrophoresis, as already performed by Bouley et al. [31].

As for the transcriptome, the approach used to identify protein biomarkers of tenderness is largely based on approaches developed by the medical community to find protein biomarkers of pathologies. It consists of comparing different samples with extreme tenderness scores (measured by mechanical measurements and/or sensory analysis) to highlight proteins whose abundance varies according to the trait studied [32,33]. This strategy has been applied in several experiments and has led to the proposal of a list of candidate biomarkers, subsequently completed by proteins revealed by bioinformatic analysis that have functional interactions with them [34,35]. Then, the relationships between tenderness and protein abundance were tested on a large number of cattle of various types (age, sex, breed) using a specifically developed immunological technique [36]. Among the 20 quantified proteins, the most confident biomarkers (four to five) were used to build equations, whose explanatory ability and error vary according to the muscle and animal type considered. For example, the variability of the shear force of the *Semitendinosus* muscle was better explained than that of the *Longissimus thoracis* muscle, which was itself better explained than the variability of its tenderness score determined by trained panelists [37]. Interestingly, the relationship between the abundance of some biomarkers and tenderness appeared to be specific to a muscle or an animal type. For instance, proteins related to fast glycolytic contractile activity were positively related to tenderness in the *Semitendinosus* muscle, but negatively in the *Longissimus thoracis* muscle of the same animal. In contrast, the Hsp70-1B was negatively related to the tenderness of these two muscles in two types of animals: young bulls from French beef breeds and Aberdeen Angus, which is known to have a more oxidative muscle metabolism than the French breeds [37]. Finally, structural proteins such as alpha-actin, F-actin-capping protein, or desmin have been identified as positive biomarkers of tenderness in different breeds and muscles by several authors [32,33].

The identification of protein biomarkers was first applied to tenderness, but is also applicable to other beef quality criteria. For example, protein biomarkers of the fat content of meat have been recently identified using the same methodology. Some protein biomarkers of tenderness also appear to be good biomarkers of other meat quality traits such as pH, color, juiciness, or flavor [38,39]. In particular, the abundance of the PRDX6 protein (peroxyredoxin), which had been identified as a biomarker of tenderness by several authors [37,40], was found to be positively related to the initial rate of p.m. pH drop and negatively to pHu in the *Longisssimus thoracis* of young Blonde d’Aquitaine bulls [38]. It was also positively related to meat redness in this same muscle. These authors also showed that the Hsp70-1B protein and the calcium-dependent protease μ-calpain were related to the color parameters: lightness, redness, and yellowness. Therefore, it is possible to develop an explanatory model based on protein abundance for most of the meat sensory qualities. Some of these proteins are also associated with muscular hypertrophy [41], suggesting joint control of the amount of meat and its sensory quality. The relationship between sensory and nutritional value has also been studied, in particular by quantification of the proteins involved in oxidative stress [42], thus opening up new opportunities for predicting the nutritional value of beef.

The first result of these studies is the considerable progress in the knowledge of the biological processes involved in determining meat tenderness [29,30]. The next step is to develop reliable tools to measure the abundance of proteins associated with tenderness to better predict this trait. Ongoing research offers hope for the rapid development of such a tool, mainly because protein quantification techniques are progressing rapidly, leading to considerable methodological simplifications [43].

## 4. Blood Biomarkers: First Encouraging Results

**Figure 1:**
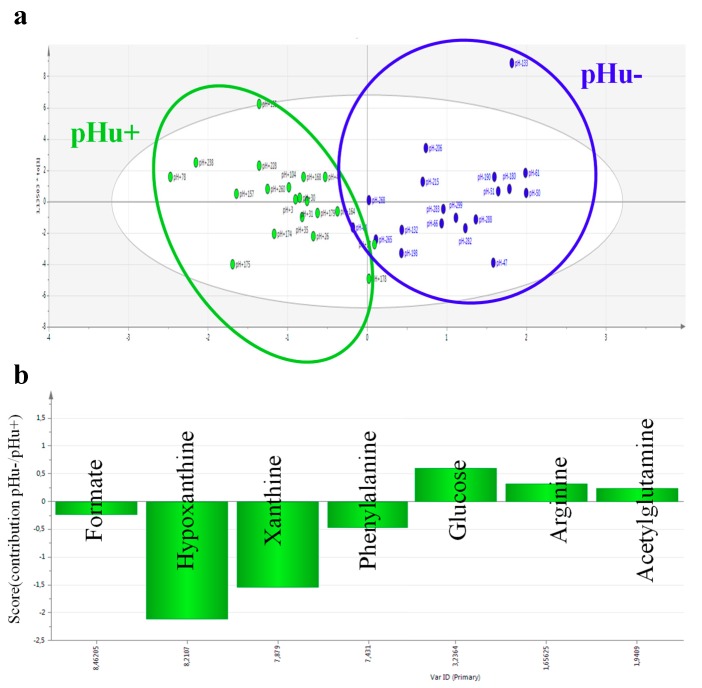
(**a**) Projection of individuals according to major principal components based on an OPLS-DA (orthogonal projections to latent structures discriminant analysis) model with an explanatory ability (R^2^Y) of 0.73 and a predictive value (Q²) of 0.64 (the pHu− and pHu + individuals are shown in green and blue, respectively); (**b**) Contribution of the seven metabolites identified by the OPLS-DA model (pHu−/pHu +). Illustration based on results published in [21].

Having confident predictive blood markers would greatly facilitate the development of phenotyping methods in live animals. The pHu+ and pHu− lines model mentioned above was used to identify blood and muscle metabolite predictors of the pHu of chicken breast meat. A first step was to analyze by high-resolution nuclear magnetic resonance or NMR (proton and phosphorus NMR for muscle, and proton NMR for serum) the muscle (breast) and serum extracts from extreme animals belonging to both lines. These analyses revealed very specific metabolomic signatures of the two groups in blood and muscle that enabled an almost perfect discrimination between them [21]. A total of 20 and 26 discriminant metabolites between the two lines were identified by multivariate OPLS-DA (orthogonal projections to latent structures discriminant analysis) in serum and muscle, respectively. Three independent models were fitted with good explanatory (R²Y) and predictive (Q²) abilities for pHu (R²Y = 0.63–0.82, Q² = 0.45–0.76). A multiblock model, including muscle and blood metabolites, was subsequently developed with even better explanatory (R²Y = 0.91) and predictive (Q² = 0.86) power. To develop a test that could be routinely used on live animals, the study focused specifically on the metabolites identified in the blood. Thus, a model including seven metabolites (acetylglutamine, arginine, formate, glucose, hypoxanthine, phenylalanine, and xanthine) always provides good discrimination (R²Y = 0.73, Q² = 0.64, Figure 1) while limiting the number of biological tests for diagnosis as much as possible. However, the predictive potential of this set of serum biomarkers must be validated on other chicken populations that are representative of the pHu variability observed in slaughterhouses and have different genetic backgrounds from the pHu+ and pHu− lines. If this validation step is successful, these biomarkers could be used in selection to exclude from parental stocks the individuals predisposed to produce high-pH or low-pH meat, or in research to evaluate innovations related to animal husbandry practices. 

In cattle, the search for plasma biomarkers of the sensory qualities of meat was initiated by proteomic analysis. In addition, bioinformatics tools have identified secreted proteins that could be good potential candidates for quantification in plasma [44]. These blood-based approaches are of considerable interest for the analysis of meat quality biomarkers in live animals. For instance, significant negative correlations were observed between blood retinol content and marbling score (*R* = −0.47, *p* ≤ 0.01), and between blood aspartic acid transaminase content and *longissimus* muscle area at the 13th rib (*R* = −0.67, *p* ≤ 0.01) in the finishing phase of Hanwoo steers [45]. Metabolomics approaches are also powerful, not only to differentiate meat from different species (cattle, pigs, and chickens), but also from different cattle breeds [46]. Furthermore, the evolution of key metabolites and associated pathways has been studied during the post-mortem aging of beef [47]. Some specific muscle metabolites have also been described to well differentiate muscles that differ in the composition of their muscle fiber types. The content of metabolites involved in the Krebs cycle change differently in different muscles according to the aging time [48].

## 5. Spectroscopic Methods: Physical Chemistry to Decipher Biology

**Figure 2:**
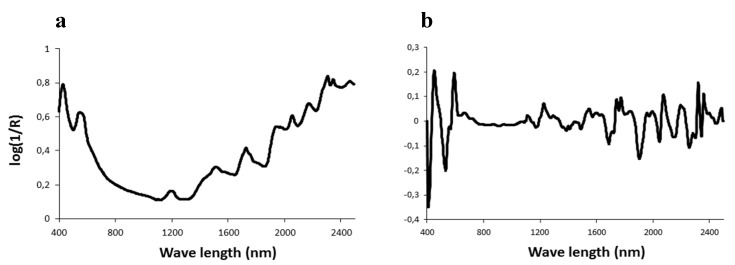
Visible/near infrared spectrum and first derivative between 400–2500 nm of a sample of bovine muscle *(Rectus abdominis)* after grinding (**a**) and lyophilization (**b**).

Spectroscopy can be defined as the study of the interaction between light and matter. Among spectroscopic techniques, near-infrared (NIR) and Raman spectroscopy are currently the most widely used techniques for predicting meat quality. The background of near-infrared spectroscopy (NIRS) was described in the review by Bertrand [49], while Raman spectroscopy was reviewed by Yang and Ying [50]. Briefly, these technologies are physical methods of analysis based on the property of light absorbed by organic molecules at specific frequencies. The relationships between the chemical composition and the absorbance values or their derivatives are then established (Figure 2).

### 5.1. Difficulties in Predicting Sensory Quality

The number of studies published in recent years and the number of companies recently equipped with NIR instruments show the importance of spectroscopic technologies and in particular that of NIRS for estimating meat sensorial quality [51,52].

The sensory characteristics of meat derive from the amount of different chemical compounds or from biological parameters (lipid content, collagen, muscle fiber typology, pH, etc. [28]). Consequently, it may be possible to predict sensorial determinations using spectroscopic methods [53,54]. However, the results reported in the literature do not allow validating this hypothesis due to different reasons: According to Liu et al. [55], in a sensory analysis, the use of a narrow scale for the intensity of sensory characteristics could reduce the precision and accuracy of their predictions. Furthermore, the samples scanned by NIRS are not exactly the same as those tested by the tasting panels. The high heterogeneity of meat traits within a muscle can contribute to generating some significant bias between the NIR-predicted values and the measured values. These observations may also explain some of the difficulties in predicting meat-eating quality using the biomarkers described above. Contrary to the studies cited above, Ripoll et al. [56] reported *R*^2^ values of 0.98 for models built to predict the tenderness of beef. These models were developed using meat obtained from different breeds and maturity levels, thus increasing the variability of the data sets used to build the models.

The prediction of shear force by NIRS or by Raman spectroscopy gives very variable results (*R*^2^ values from 0.01 to 0.74 [57,58,59,60,61,62] (Table 2). Elsewhere, Liao et al. [63] reported R^2^ values in calibration of 0.72, whereas the R^2^ value in validation was only 0.27. Other authors have shown that NIR is able to predict the tenderness of pork after cooking with similar variability to that shown above (*R²* = 0.20 to 0.72 [63,64]).

The on-line prediction of *Longissimus lumborum* tenderness in slaughterhouses by NIRS was tested by Rust et al. [65]. They observed that the proportion of muscles correctly classified as tender was 70%. According to Leroy et al., Liu et al., and De Marchi et al. [55,59,66], these results could be explained not only by the variability of samples and data sets, but also by the variability within replications for the Warner–Braztler shear force method [67,68]. De Marchi et al. [68] also reported some changes in the spectral information of the infrared segment due to the modification of the muscle structure by the impact of grinding. Consequently, the R^2^ values of the models were not improved by the effect of grinding, and the values of this statistic may even deteriorate at times.

**Table 2:** Statistical parameters of Warner–Braztler shear force (WBSF) and tenderness prediction in meat by visible/near-infrared (VIS/NIR) and Raman spectroscopy.

Method	Meat	Parameter	R^2^c	SEc	R^2^cv	SEcv	R^2^p	SEp	Reference
VIS/NIR (R)	beef	WBSF	0.72	0.84					[55]
VIS/NIR (R)	beef	Tenderness	0.98	0.37			0.98	0.35	[56]
		WBSF	0.74	0.66			0.74	1.06	
NIR (R)	beef	WBSF	0.65	2.30	0.53	2.67			[57]
NIR (R)	beef	WBSF	0.21	0.48					[58]
NIR (T) (intact)	beef	WBSF			0.31	3.07			[68]
NIR (T) (ground)	beef	WBSF			0.12	3.48			
VIS/NIR (R) (intact)	beef	WBSF			0.34	9.39			
VIS/NIR (R) (ground)	beef	WBSF			0.13	10.74			
Raman	beef	WBSF	0.94	2.00	0.79	3.90	0.23	8.80	[60]
Raman		WBSF			0.75	0.63			[61]
		Tenderness			0.65	0.97			
Raman	lamb	WBSF			0.06	13.60			[62]
VIS/NIR (R) (intact) on line	pork	WBSF	0.72	0.23			0.27	0.36	[63]
VIS/NIR (R) (ground)	pork	WBSF	0.48	4.22	0.30	4.98	0.25	5.51	[64]
NIR (R)	beef	WBSF	0.45	9.32		10.00			[67]
NIR (R)	beef	WBSF	0.17	15.69		15.89			
NIR (R)	beef	WBSF			0.25	11.19			[66]
NIR (T)	beef				0.41	9.59			
NIR (R) freeze dried	beef	WBSF	0.20	4.65	0.12	4.99			[59]
NIR (R) fresh minced	beef	WBSF	0.08	5.09	0.03	5.21			

VIS/NIR (R): Visible/near infrared in reflectance. VIS/NIR (T): Visible/near infrared in transmission. R^2^c: Coefficient of determination of calibration. SEc: Standard error of calibration. R^2^cv: Coefficient of determination of cross-validation. SEcv: Standard error of cross-validation. R^2^p: Coefficient of determination of prediction. SEp: Standard error of prediction.

In conclusion, the use of spectroscopic techniques is not fully effective in predicting the sensory quality (tenderness or shear force) of meat. The high variability of the results obtained can be partially explained by the low repeatability of the reference method measurements, as with any predictive method (including the search for biomarkers above). However, some promising results indicate that further research is needed to obtain suitable models for predicting the sensory quality of meat.

### 5.2. Routine Uses for Nutritional Quality

Although it is still difficult to predict the sensory quality of meat using NIRS, this technology is nonetheless recognized and used to determine the chemical composition and therefore the nutritional value of meat.

For instance, the lipid content of chicken or duck breast (from lean or fat ducks) can be routinely measured by NIRS (replacing chemical methods) thanks to the development of very robust prediction equations. In chickens, the coefficient of determination (*R*^2^) is between 0.8–1.0, and calibration standard deviations are close to 0.2 (*R*^2^ = 0.98, [69,70], *R*^2^ = 0.83, [71]); in ducks, an *R*^2^ of 0.94 and a standard deviation of 0.31 were published [72]. The fatty acid composition is an important trait that can influence the nutritional, sensory, and technological quality of meat. It is possible to estimate by NIRS technology the content of the main fatty acids and their different types (polyunsaturated, monounsaturated, and saturated) with excellent coefficients of determination (>0.9 [70] for freeze-dried samples, although those obtained on thawed samples are lower (approximately 0.6 [73]). The protein and dry matter content of chicken meat can also be estimated by NIRS on freeze-dried samples using models with high *R*^2^ values (approximate values of R^2^ = 0.98 and standard deviation = 0.2 [70]). In chicken, NIRS models constructed from thigh muscles (fatter and more variable than breast muscles) to predict chemical composition have even higher R^2^ values than those developed from breast meat [74].

In the case of pork, many studies have focused on predicting its chemical composition. Calibration for predicting intramuscular fat content was of variable quality, with R² values ranging from 0.28 to 0.88. The error was close to 1% regardless of the type of sample, i.e., intact meat, crushed or salted/dried meat [64,75,76,77,78]. The composition of polyunsaturated, monounsaturated, and saturated fatty acids of backfat can also be predicted by NIRS with good precision (*R²* = 0.61 to 0.99) [79,80,81,82]. The accuracy is roughly comparable regardless of the type of fatty acid, with an error of about/approximately 1%. However, the quality of the prediction by NIRS in backfat depends on the content of the various fatty acids in the product: the relative error is less than 5% when the saturated and monounsaturated fatty acid content is high, while it is close to 10% when the polyunsaturated fatty acid content is high. It is also possible to predict the fatty acid profile of the intramuscular fat in Iberian pork loin with very good precision (*R²* > 0.99 and error of about 1%) for the prediction of polyunsaturated, monounsaturated, and saturated fatty acids [83]. Promising results in predicting the IMF content and fatty acid composition of pork from various local breeds were also obtained by Bozzi et al. [84], using the Fourier transformation NIRS technology (FT-NIRS). This technology improves the signal-to-noise ratio, spectral resolution, and wavelength accuracy, and reduces scan time [85]. Partial least square regression models were established and validated on external data using FT-NIRS. At the validation step, high determination coefficients and low errors were obtained for IMF content (R² ≥ 0.96, root mean square error (RMSE) ≤ 0.66) and polyunsaturated fatty acid proportion (*R²* ≥ 0.87, RMSE ≤ 0.70). Performance was slightly lower, but still valuable for monounsaturated fatty acid (*R²* ≥ 0.77, RMSE ≤ 1.13), and saturated fatty acid proportions (*R²* ≥ 0.77, RMSE ≤ 0.97) [84]. Thus, FT-NIRS seems promising to estimate the principal parameters of fatty acid groups on pig muscle samples and therefore the nutritional composition of pork.

### 5.3. High Expectations for the Technological Quality of Meat

The value of NIRS technology to evaluate the technological properties of meat has been mainly studied in pork and chicken. In chicken, the best predicted criteria are color indexes (redness and yellowness) with correlation coefficients greater than 0.8 [86,87]. However, the interest of using NIRS to predict these parameters is limited, because it is very easy to measure them by spectrometry. The prediction performance for pHu, drip loss during storage, and cooking losses is generally lower, with correlation coefficients between 0.6–0.8 [70,86]. However, these levels of correlation suggest prospects for improving these characteristics. This is not the case for other traits such as pH15 min p.m. or shear force [86,87], for which correlation coefficients <0.5 have been obtained.

The selection of raw meat according to its technological potential is now widespread in the pork industry. However, sorting as it is practiced today (mainly based on the pHu value) only covers certain aspects of meat quality, and lacks precision in industrial conditions. Rapid acquisition alternatives such as NIRS or vision/hyperspectral imaging show strong potential for predicting quality. The majority of the studies focusing on predicting the pHu have reported calibrations with *R²* values between 0.65–0.87 [64,77,88,89]. However, the pHu prediction error remains high (0.05 to 0.18) compared to the repeatability of the reference method (0.03). Numerous studies show that it is also possible to establish calibrations that are sufficiently accurate to predict the water retention capacity of meat. This is the case for drip loss, for which R² values between 0.31–0.76 (often around 0.60) have been reported [75,77,78,88,90,91,92,93]. However, the prediction error of drip loss remains high, between 1% and 2%. NIRS prediction of the processing yield has also been studied in pork. The few publications available show satisfactory accuracy for ham and loin (*R²* = 0.57 to 0.78) [94,95].

Vision and hyperspectral imagery are currently considered as high-potential technologies for the slaughter/processing sector. Vision systems are automatic alternatives to measure meat color, using a camera (contactless) rather than a colorimeter, which requires a contact probe and an operator. The camera has to be previously calibrated to obtain a reliable color measurement, and the RGB camera signal is converted to L*a*b* color space using colorchecker tiles. Before obtaining the L*a*b* value of the meat, the images must be processed to extract the color of a specific region of interest (ROI) from the meat. Hyperspectral systems use a very similar approach, but a NIR spectrum is obtained for each pixel, so after obtaining a mean spectrum on a specific ROI, classical chemometrics are applied to perform prediction models, such as NIRS. Compared to NIRS, vision and hyperspectral imagery have the advantage of a contactless measurement, and can be easily integrated into a production line because they do not require operator intervention. The fields of application are the same as for NIRS, although there are currently not enough publications to draw conclusions regarding the accuracy of these techniques. Properly adjusted vision calibrations have been established to predict the ultimate pH of pork (*R²* = 0.49 to 0.72) [96,97], while the prediction of the processing yield of ham by this same technique lacks sufficient accuracy to be operational (*R²* = 0.31 to 0.43). Hyperspectral analysis, which can be considered as the combination of vision and NIRS, may be useful for predicting drip loss (*R²* = 0.60) [97] and for classifying meat according to its technological quality. For example, 84% of pork was correctly classified as PSE, RFN (red, firm and non-exudative), and RSE (red, soft, and exudative) meat [98]. This still undeveloped prediction technique will require further work to assess its relevance in predicting the quality of meat from different species.

## 6. Development of Phenotypic Models for Beef Evaluation

### 6.1. Principles

The Australian beef industry and researchers have jointly built, as part of a common and collective strategy, the MSA (Meat Standards Australia) grading scheme, which is a mathematical model for predicting the eating quality of beef for each “muscle × cooking method” combination. This model was constructed from a large database of consumer tests using a standard protocol [99,100]. A dozen parameters with a statistically significant effect on eating quality, such as traits characterizing animals (physiological maturity, weight, genetic type, sex, etc.), pre-slaughter and slaughter conditions (carcass hanging method, etc.), meat (pH, color, marbling, etc.), and post-mortem events (aging time, cooking method, etc.) are considered in the model as well as the interactions between them. Some stakeholders of the beef industry are already using this model, and it appears that its use has contributed to reducing the decline in beef consumption in Australia.

**Figure 3:**
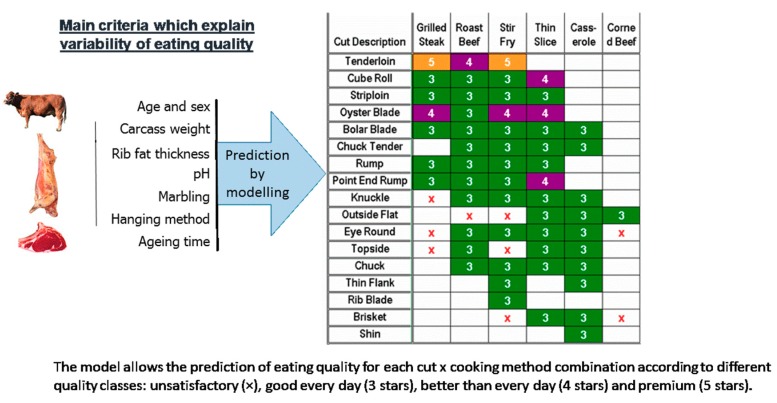
Prediction of the overall beef eating quality score (combining tenderness, flavor liking, juiciness, and overall liking) from different traits related to animals, carcasses, and cuts using the “Meat Standards Australia” (MSA) grading scheme.

In practice, the slaughterhouse is the backbone of the system. A specific grader who is accredited after training, and receives recurrent trainings, grades the carcasses. Then, the MSA model predicts an overall quality score called MQ4 (for “Meat Quality 4”) on a scale of 0 to 100, for each piece of meat associated with a specific cooking method and aging time. This score is a linear combination of consumer scores for tenderness, flavor liking, juiciness, and overall liking. In sensory testing, to make the best link with the quality ranking of meat also given by consumers, four quality classes are used: unsatisfactory, good every day (3*), better than every day (4*), and premium (5*) (Figure 3). The values of the overall MQ4 score defining the limits between each quality class are precisely calculated for each data set and regularly refined: they are about 40 (between unsatisfactory and 3*), 60 (between 3* and 4*), and 80 (between 4* and 5*) on a scale of 0 to 100 [99,100].

### 6.2. Applications

The principle of the MSA system has been evaluated in various countries such as South Korea, the USA, Japan, South Africa, New Zealand, Northern Ireland, Poland, France, and the Republic of Ireland [101,102,103,104,105,106]. The general conclusion is that the MSA methodology is relevant in all these countries. However, the relative weighting coefficients for tenderness, flavor liking, juiciness, and overall liking in the optimal calculation of the MQ4 score vary slightly between countries, and the optimal limits between quality classes can be refined for each country or each group of consumers.

In the French context, experts have judged it to be rigorous, relevant, and credible. Experts have recognized that the MSA system has helped to federate a large number of Australian professionals and scientific stakeholders. This approach, based primarily on real consumer satisfaction, is likely to upset the traditional attitudes and political positions of stakeholders in the beef sector. We should not consider the MSA system as a new official quality label, but rather as a rigorous tool to better use the existing official quality signs [107]. Two studies [106,108] experimentally tested the MSA system in France. They concluded that the MSA system provides a fairly good prediction of the eating quality of French beef, despite differences in animal type (cows and young bulls in France versus steers and heifers in Australia) and the degree of cooking (55 °C for “rare” cooking in France versus 74 °C for “well-done” meat in Australia). Using the MSA system, about 70% of the French meat was correctly ranked according to the different classes (unsatisfactory, 3*, 4*, or 5*). This rate is as good as if not better than that observed in other countries where the MSA system has been studied, including Australia. Prediction using the MSA system is even better than prediction methods based on muscle biochemistry [109] or genomic data [110].

In the case of a binary use of the MSA system in Europe (i.e., unsatisfactory quality versus acceptable quality with no distinction between classes 3*, 4*, or 5*), the probability of really disappointing the consumer—that is to say, erroneously classifying a poor quality sample as 3* or greater, is only 7% based on data from different European countries (Northern Ireland, Ireland, France, and Poland). Considering that currently, according to the same data set, the sensory quality of about 25% of meat is deemed unsatisfactory, a binary use of the MSA system would therefore constitute an important step forward, as it could theoretically reduce customer dissatisfaction from 25% to 7% [111]. This is particularly important for an expensive product such as beef. A European beef quality assurance system similar to MSA would need to be simple, effective, and sufficiently flexible to allow companies to develop their own brands [112]. Furthermore, it was proposed to consider in the European model both the gender (female, castrated male, or entire male) and breed type (dairy or meat breed), because recent work has shown that their effects on the sensory quality of beef are not fully explained by animal age or carcass characteristics (weight, fatness, etc.). It was also suggested to assess the physiological maturity of animals that are included in the MSA system using the degree of carcass ossification (as in Australia) for young animals and the age of animals (as in Europe) for older ones [113]. All these improvements would certainly help to increase the accuracy of the prediction of beef eating quality in Europe. Further research will be conducted in these directions as part of the activities of the International Meat Research 3G Foundation (https://imr3gfoundation.org/) recently launched under the auspices of the United Nations Economic Commission for Europe (UNECE).

### 6.3. Perspectives

Carcass quality criteria (i.e., conformation and fat scores based on the EUROP grid) according to which farmers are paid do not have a clear and systematic relationship with the eating quality of beef [114]. This partly explains why a consumer can buy very expensive beef without necessarily being satisfied, and vice versa [115]. In general, consumers are willing to pay more if they are sure that the product will be of higher quality. Therefore, one can expect that premium products are likely to generate significant profits compared to “good for everyday” products, regardless of the market. However, this difference may vary from country to country, French and Japanese consumers being the most likely to pay more for premium quality products [111,116]. In Australia, implementation of the MSA system has generated significant profits that were distributed to various stakeholders in the sector: producers, slaughterers, and others. It was calculated that $12.50 of additional revenue was generated for every dollar invested over five years (2010/2011 to 2014/2015). Therefore, there is a real financial incentive in Australia to produce premium-eating quality meat, which is not yet the case in many countries that continue to pay producers according to carcass characteristics, i.e., conformation and fatness [117].

The MSA prediction model also opens up new possibilities for animal breeding. The potential of a muscle to produce beef of a predicted quality, weighted by the relative weight of this muscle in the carcass, makes it possible to calculate a global MSA index of sensory quality for the carcass [118]. This MSA index can be considered as a new phenotype to evaluate the animal’s potential to produce meat of a given eating quality. This index could potentially be introduced into genetic selection schemes to include the sensory quality potential of animals, which has never been achieved so far.

## 7. Advances and Barriers to the Development of Predicting Tools

The meat sector is significant in Europe. However, it is facing a difficult economic context, particularly due to a steady decline in per-capita meat consumption, especially red meat. The reasons for this decline in consumption are numerous, and include the high variability of sensory quality as mentioned in this article as well as criticism of the nutritional qualities. These qualities, particularly tenderness in cattle, are sometimes or often considered insufficient by professionals or consumers. All these observations fully justify the analytical or technological research carried out on the prediction of these quality components. However, the quality of the predictions of the different quality criteria is often modest. In addition, operators in the meat sector, from livestock to processing, have small profit margins, and are therefore greatly concerned about the economic profitability of their activities. For cost/benefit reasons, they are reluctant to incorporate certain advances in research and development into their practices, which is noted and regretted by researchers [119]. Unfortunately, the economic crisis mentioned above is likely to reinforce this phenomenon.

In addition, it is clear that marketing channels are increasingly complex, disconnected from the animal and carcass, and include many intermediaries between the producers and consumers. Consumer behavior is also changing in terms of the place of purchase (less and less in the butcher’s shop and more and more in supermarkets) and consumption (more away from home), the nature of the products consumed (more elaborate products or individual portions), and in terms of expectations, which have diversified over the years, now including social concerns related in particular to animal welfare and protection of the environment [120]. Thus, consumer preferences, behavior, and their perception of meat and meat products are heterogeneous and depend not only on the appearance and eating qualities of the meat, but also on psychological and marketing aspects [121]. Therefore, research and development (R & D) must broaden its scope [122]. While research activities have largely focused on the intrinsic characteristics of meat, which are the sensory, technological, nutritional (subject of this article), and health qualities, the extrinsic qualities associated with the product, which meet broad societal expectations, must now be taken into account in interaction with the former [2].

In general, research directly related to consumer expectations is increasingly necessary [123], particularly to objectively predict the intrinsic qualities of meat, as well as its extrinsic qualities [124]. However, research and development are generally carried out over a long time scale, which may not be compatible with the short-term concerns of professionals [119]. At the same time, scientists must be able to take into account the expectations of professionals and consumers in order to guide their work as effectively as possible and to encourage the appropriation of technological innovations by the stakeholders in the sector.

There is also a pressing need for innovation with regard to trading, especially for export. In this perspective, the Australian methodology (the MSA system) is promising, and could become an international standard under the United Nations Economic Commission for Europe (UNECE) [125]. The search for biological predictors of quality, combined with recent technological evolutions, makes it possible to envisage practical applications in the medium term. It is undeniable that all of this research has led to considerable advances in the knowledge of the genetic and biological mechanisms governing the establishment of the different components of meat quality [126]. This improved knowledge may contribute to the development of selection tools (such as the genetic test of meat coloring developed for chicken) as well as decision support tools to propose innovative production strategies adapted to the biological potential of animals and the production objectives of the various sectors. For example, the development of spectral methods to authenticate the animal feed of animals or qualify meat in terms of nutrition is also promising. In order to be routinely applicable in a professional context, the diagnosis tool must be easy to use, give a result quickly after sampling, and have a limited cost. Some methods developed so far do not yet meet all these criteria [126].

Finally, this review also opens up new perspectives on the possibility of combining different types of the above-mentioned technologies. So, could we consider looking for biomarkers of phenotypes based on spectral methods? Similarly, would there be any interest in including in the MSA model some biological predictors resulting from high-throughput molecular approaches or spectral-based methods? This integration work remains to be done, but should undoubtedly contribute to the improvement of meat quality prediction tools and the subsequent application thereof.

## 8. Conclusions

Numerous technological innovations based on genomics or modeling approaches, or spectral or physical methods, have been described in this article to predict the specific intrinsic qualities of meat such as sensory (tenderness, flavor), technological (defects related to tissue integrity, ultimate pH, processing yield), or nutritional (lipid content, fatty acid composition) traits. Most of these approaches require additional work and methodological developments to be routinely applicable. However, their ability to analyze a large number of samples at a reduced cost, as well as their ability to predict the desired quality criteria, are fundamental elements for attracting the interest of the stakeholders of the sector. The appropriation of these methodologies by researchers is a key issue, and some laboratories already value these tools as part of their work on the impact of production factors on the quality of meat. Appropriation by the professionals will occur in a second phase and require more dialogue between professionals and researchers to properly define the objectives to be achieved as well as the conditions required for developing these tools (in terms of cost/benefit ratio in particular). In addition, research must also focus on integrative approaches to comprehensively predict all the desired quality criteria, which implies the combination of innovations described in this article on the one hand, but also insertion thereof into a more global thought process that includes a sociological dimension of research questions. The ultimate goal is to respond better to the expectations of industrials and consumers through better appropriation of innovations by the industry.

## Figures and Tables

**Table 1:** Studies dedicated to the search for gene biomarkers of meat quality in pork and chicken.

Species	Meat	Animal Model	Parameters	Reference
Pork	Ham	Normal and defected (destructured) groups within genotype	Destructured ham	[6]
Pork	Loin	Low and high-IMF groups within genotype	IMF	[7,8]
Pork	Loin	Low and high-WBSF groups within genotype	WBSF	[9,10,11]
Pork	Loin	Gradual variability of meat quality using two breeds produced in different farming systems	pHu, color, drip loss, IMF, WBSF, tenderness, and juiciness	[12,13]
Pork	Loin	Gradual variability in meat quality using commercial pigs (Duroc × Landrace × Yorkshire)	pHu, color, drip loss, IMF, WBSF, tenderness, and juiciness	[14]
Pork	Loin	Gradual variability of meat quality using two breeds produced in different farming systems	Meat quality index combining several technological and sensory parameters	[15]
Chicken	Breast	Lean and fat experimental lines	pHu	[19]
Chicken	Breast	F2 cross between the lean and fat experimental lines	pHu	[20]
Chicken	Breast	Low and high-pHu experimental lines	pHu	[22]
Chicken	Breast	Experimental slow-growing line	Color	[23]
Chicken	Breast	Low and high-pHu experimental lines	pHu	[27]

IMF: intramuscular fat content. pHu: ultimate pH. WBSF: Warner–Bratzler shear force.
